# Effects of Particle Size Distribution with Efficient Packing on Powder Flowability and Selective Laser Melting Process

**DOI:** 10.3390/ma15030705

**Published:** 2022-01-18

**Authors:** Zachary Young, Minglei Qu, Meelap Michael Coday, Qilin Guo, Seyed Mohammad H. Hojjatzadeh, Luis I. Escano, Kamel Fezzaa, Lianyi Chen

**Affiliations:** 1 Department of Mechanical and Aerospace Engineering, Missouri University of Science and Technology, Rolla, MO 65409, USA; zay7c4@umsystem.edu (Z.Y.); mmcckd@mst.edu (M.M.C.); 2 Department of Mechanical Engineering, University of Wisconsin-Madison, Madison, WI 53706, USA; mqu22@wisc.edu (M.Q.); qguo46@wisc.edu (Q.G.); hojjatzadeh@wisc.edu (S.M.H.H.); escanovolque@wisc.edu (L.I.E.); 3 Department of Materials Science and Engineering, University of Wisconsin-Madison, Madison, WI 53706, USA; 4 Advanced Photon Source, Argonne National Laboratory, X-ray Science Division, Lemont, IL 60439, USA; fezzaa@aps.anl.gov

**Keywords:** flowability, particle size distribution, additive manufacturing, powder bed fusion

## Abstract

The powder bed-based additive manufacturing (AM) process contains uncertainties in the powder spreading process and powder bed quality, leading to problems in repeatability and quality of the additively manufactured parts. This work focuses on identifying the uncertainty induced by particle size distribution (PSD) on powder flowability and the laser melting process, using Ti6Al4V as a model material. The flowability test results show that the effect of PSDs on flowability is not linear, rather the PSDs near dense packing ratios cause significant reductions in flowability (indicated by the increase in the avalanche angle and break energy of the powders measured by a revolution powder analyzer). The effects of PSDs on the selective laser melting (SLM) process are identified by using in-situ high-speed X-ray imaging to observe the melt pool dynamics during the melting process. The results show that the powder beds made of powders with dense packing ratios exhibit larger build height during laser melting. The effects of PSD with efficient packing on powder flowability and selective laser melting process revealed in this work are important for understanding process uncertainties induced by feedstock powders and for designing mitigation approaches.

## 1. Introduction

The powder bed-based additive manufacturing process makes parts through fusing or binding powders in a powder bed [1,2,3,4]. Unfortunately, the resulting part quality is not as reliable or repeatable as that made by conventional mature manufacturing methods. One cause of non-uniformity of part quality is variation of powder size distribution (PSD). Uncertainty in PSD of feedstock powders leads to reduction in quality and repeatability for powder spreading [5,6,7].

Previous works have investigated the effects of varying powder size distribution on the resulting material properties [1,6,8,9,10,11]. Variations in PSD have been found to have effects on the resulting physical, surface, and mechanical properties [5,6,12,13,14,15,16,17]. Previous research results have also shown that changes from a homogenous powder to non-homogeneous powders result in an overall reduction of powder flowability [18,19]. Some representative works are summarized below.

Liu et al. conducted work highlighting the effects of isolating two PSDs: a narrow, near-homogenous, PSD and a wider, heterogeneous, PSD with near identical average powder size [20]. Liu’s work concluded that changes to PSD affect powder bed flowability, part density, hardness, and surface finishes. Work by Meier et al. utilized discrete element method (DEM) to study the frictional, rolling, and cohesive forces of powders and their effects on adhesion and uniformity of various powders [1]. Work by Ma et al. tested a wide range of PSD of alumina powders to investigate the finalized sintering characteristics [14] and observed increases in densification rates and grain growth through broadening of the PSD during laser sintering. A recent work by Bai et al. identified the effects of PSD on binder jetting additive manufacturing using a bimodal powder mixture to obtain high-density powder beds [21].

Previous works, however, have not determined the causes of sudden changes in flowability during SLM process that led to failures during part manufacturing.

This work uses a particle dense packing model (described in the references [11,22]) to design PSDs with efficient packing to determine the effect of the dense packing on the flowability and SLM dynamics. The flowability of powders with different PSDs are tested using the powder revolution method; the SLM dynamics of the powder bed made of powders with different PSDs are tested utilizing high-speed X-ray imaging. The results show that the flowability and SLM dynamics exhibit a sudden change at the PSDs with dense packing.

## 2. Materials and Methods

### 2.1. Powders

**Figure 1:**
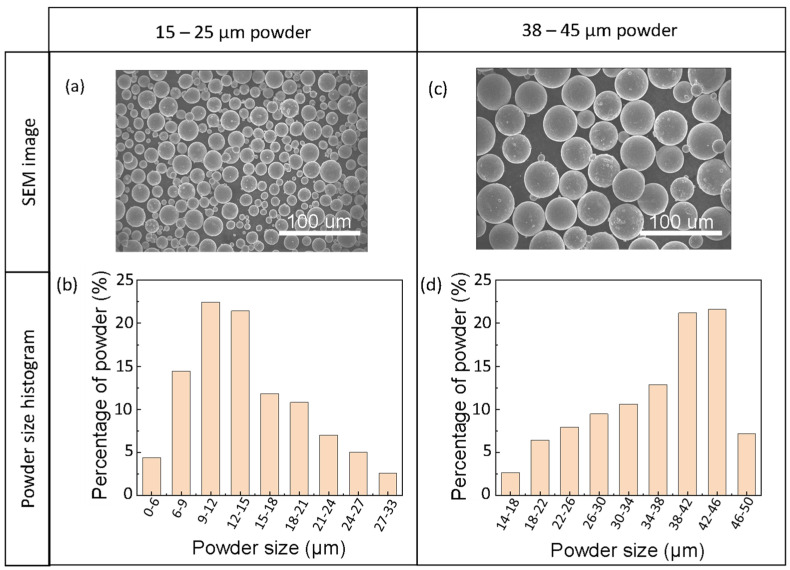
Morphology and size distribution of powders. (**a**) SEM image of 15–25 μm powder. (**b**) SEM image of 35–48 μm powder. (**c**) Particle size distribution of 15–25 μm powder. (**d**) Particle size distribution of 35–48 μm powder. The percentage used in (**b**,**d**) is number percentage.

Two plasma atomized powders from Pyrogenesis (Montréal, QC, Canada) were used in this study. The size specifications are ~15–25 μm (small) and ~38–45 μm (large) according to manufacturer’s specifications. Scanning electron microscope (SEM) imaging was conducted to ensure the powder size lied within $D_{50}$ specifications. Testing looked at hundreds of individual powders within multiple SEM images to determine the histogram of the particle size distribution provided in Figure 1.

**Figure 2:**
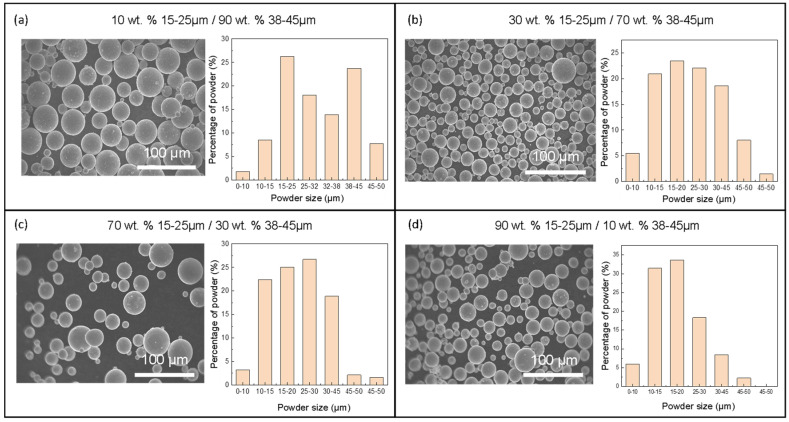
Morphology and size distribution of mixed powders. (**a**) 10 wt.% 15–25 μm powder + 90 wt.% 38–45 μm powder. (**b**) 30 wt.% 15–25 μm powder + 70 wt.% 38–45 μm powder. (**c**) 70 wt.% 15–25 μm powder + 30 wt.% 38–45 μm powder. (**d**) 90 wt.% 15–25 μm powder + 10 wt.% 38–45 μm powder.

The 15–25 μm powder and 35–48 μm were mixed to create four additional particle size distributions. Mixing was conducted by a Turbula mixer (WAB, Allendale, NJ, USA) to ensure proper mixing. The four additional powders were set based on weight percentages of small powder mixed with larger powder at 10, 30, 70, and 90 percent. The newly mixed powders were observed under SEM and an analysis of hundreds of individual powders were conducted to generate the histogram of the particle size distribution shown in Figure 2.

Work conducted by Sinico et al. and other research articles utilize mathematical representative models such as size distributions to give an accurate model for the PSDs [23]. The $D_{10},\;D_{30},\;D_{50},\;D_{70},$ and $D_{90}$ distributions were calculated for the six PSDs (Table 1).

### 2.2. Efficient Particle Packing

**Figure 3:**
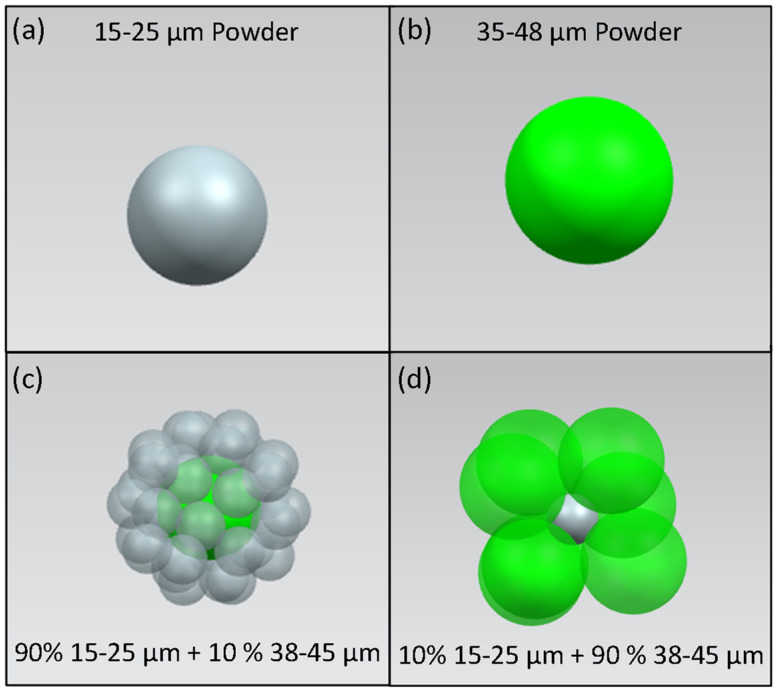
A 3D representation of particle packing model. (**a**) 15–25 μm powder. (**b**) 38–45 μm powder. (**c**) 15–25 μm powders packed around a central 38–45 μm powder. (**d**) 38–45 μm powders packed around a central 15–25 μm powder. The first packing model has the greatest packing efficiency.

The particle size distributions at 10 and 90 percent of small powder mixed with large powder were created to generate the highest packing density with the powder sizes. The ratio is explained by particle dense packing model [11,22] to generate the highest density of surrounding particles around a central particle, as shown in Figure 3.

To determine the packing density, the average powder size is used. The final weight percentages were rounded to the nearest 10%. The powder ratio was determined depending on the size ratio R of the central powder $r_{i}$ to the surrounding powder $r_{j}$. Three equations are utilized in the determination of powders surrounding a central powder according to ratio *R*:

(1) $$N^{T}=\{\begin{array}{ll} \frac{4\pi }{6\arccos\left\{\sin\left(\frac{\pi }{3}\right){\left[1-\frac{1}{{\left(R+1\right)}^{2}}\right]}^{1/2}\right\}-\pi } & \mathrm{for}\;0.225\leq R<0.414,\\ \frac{4\pi }{8\arccos\left\{\sin\left(\frac{\pi }{4}\right){\left[1-\frac{1}{{\left(R+1\right)}^{2}}\right]}^{1/2}\right\}-2\pi } & \mathrm{for}\;0.414\leq R<0.902,\\ \frac{4\pi }{10\arccos\left\{\sin\left(\frac{\pi }{5}\right){\left[1-\frac{1}{{\left(R+1\right)}^{2}}\right]}^{1/2}\right\}-3\pi } & \mathrm{for}\;R\geq 0.902. \end{array}$$

The $N^{T}$ is the number (including full powder and partial powder) of $r_{j}$ powders that can be theoretically placed around the central $r_{i}$ powder. The equation can be simplified to determine the integer amount N of surrounding powders $r_{j}$ capable of being placed around the central powder $r_{i}$. The resulting relationship is converted to weight percentages of the small and large powders. Additional information regarding the methodology to determine the mixing ratios with dense packing can be found within the works of Miracle et al. [11,22].

### 2.3. Flowability Testing

**Figure 4:**
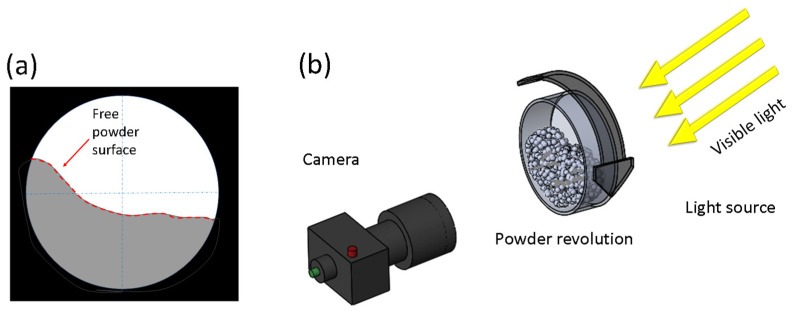
Schematic of powder revolution testing. A Mercury Scientific revolution powder analyzer is used for the analysis of flowability properties of powder with varying particle size distributions. The free powder surface (**a**) is tracked under hundreds of revolutions to capture and quantify flowability characteristics. The analyzer uses a light source and visible light camera to capture events during powder revolution (**b**). Captured images are used to determine avalanche angle and break energy of the powders.

Powder revolution testing was conducted to determine the flowability characteristics using a Mercury Scientific revolution powder analyzer (Newtown, CT, USA). Flowability characteristics of break energy and avalanche angle were determined. All tests were done with a single batch of powder for multiple iterations and averaged to determine the flowability characteristics. The powder analyzer uses a visible light camera to capture the powder dynamics during rotation, as shown in Figure 4.

Flowability testing required the creation of ~250 g of each powder sample for testing. The powders were mixed with a Turbula mixture. After mixing, the powders were placed within the cylindrical testing drum inside the Mercury powder analyzer. Vibrations were applied to identify changes in the effective density recorded at one to two second intervals. The density change monitoring is done by capturing volume changes of the powders with known mass using the system’s visible light camera. Once vibrations are completed, the cylindrical testing drum rotates. The system captures the angle of the powder surface relative to horizontal direction. After hundreds of avalanches, the system calculates the average flowability characteristics of the powder system.

Two flowability characteristics, avalanche angle and the break energy, were used to evaluate the flowability of powders. The effectiveness of avalanche angle and break energy to evaluate powder flowability has been demonstrated in previous publications by Nalluri et al. [24] and Hancock et al. [25]. The avalanche angle is the average angle of the powder buildup along the cylindrical wall just before an avalanche occurs during revolution. The break energy is defined as the difference between the maximum energy of the sample powder before the avalanche begins and the initial energy of the powder sample before rotation begins. The total powder’s potential energy is determined using the location of the pixel relative to the bottom of the drum for reference. The potential energy $U_{pixel}$ of a pixel is defined as

(2) $$U_{pixel}\left(\mathrm{mJ}\right)=M_{pixel}\left(\mathrm{kg}\right)*H_{pixel}\left(m\right)*G\left(\frac{m}{s^{2}}\right)*1000.$$

where the height $H_{pixel}$ of each pixel is multiplied by the gravitational constant and mass of the individual pixel. The mass of a pixel $M_{pixel}$ from the visible light camera is defined as

(3) $$M_{pixel}=\left(\frac{M_{total}}{V_{total}}\right)*V_{pixel}$$

where $V_{total}$ and $V_{pixel}$ are the volumes of the total powder system and individual pixel, respectively. The cumulative total of the powder and its location within the cylinder defines the total potential energy of the powder system.

The static density of the powders was characterized by the apparent density and tapped density. The apparent density was measured by a Mercury Scientific powder revolution analyzer before applying any vibration. The tapped density was determined using a built-in system within the powder revolution analyzer after roughly 7.5 min of cyclical vibration. The Hausner ratio was calculated as the ratio of the tapped density to the apparent density of the powder sample. The apparent density, tapped density and Hausner ratio of the powders are shown in Table 2 below.

Density testing results show that the highest apparent density is 2.5 g/cc at 100% 15–25 μm powder. The greatest tapped density occurs at 10% 15–25 μm powder with a density of 2.74 g/cc. The greatest Hausner ratio is 1.14 at 90% 15–25 μm powder. Generally, a high Hausner ratio indicates a low flowability. The samples with 10% and 90% 15–25 μm powder were designed to have dense packing ratios and exhibit the greatest effects on the tapped density and Hausner ratios, respectively.

### 2.4. In-Situ Characterization of Selective Laser Melting Dynamics

In-situ high-speed X-ray imaging was used to determine the effects of particle size distribution on selective laser melting dynamics. Testing is conducted with a high-flux synchrotron X-ray with a first harmonic energy of 24 keV and an energy bandwidth of 5–7% (Beamline 32-ID-B, Advanced Photon Source, Argonne National Laboratory, Lemont, IL, USA). A scintillator (LuAG:Ce, 100 µm thickness) is used to capture the X-ray signal where the signal is converted into visible light and recorded by a high-speed camera (FastCam SA-Z, Photron, Tokyo, Japan) [26]. A frame rate of 50 kHz and an exposure time of 1 µs was used to capture the laser melting dynamics. The field of view for the X-ray is 768 × 512 pixels with a resolution of ~2 µm per pixel. The laser beam size, power, scan speed and scan length are 90 μm, 364 W, 0.9 m/s and 2.5 mm, respectively. The typical sample assembly, which is composed of a miniature Ti6Al4V metal substrate with a thickness of about 0.40 mm, a height of about 2.95 mm, and a powder layer thickness of about 100 µm, is sandwiched between two pieces of glassy carbon, which is transparent to the incident X-ray beam. The test sample is placed in a vacuum chamber filled with 1 atm high purity argon gas to prevent oxidation during laser melting.

**Figure 5:**
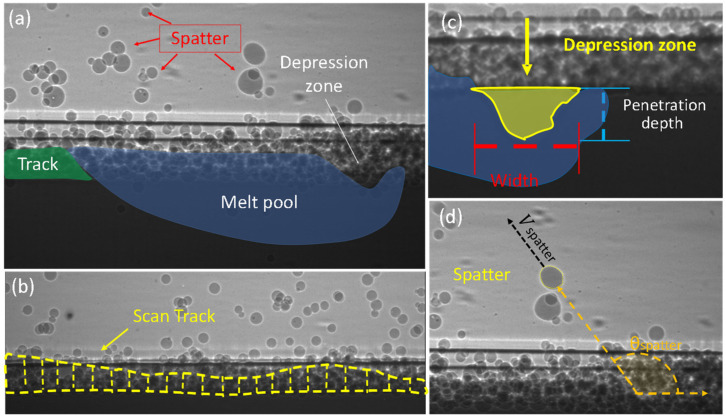
Selective laser melting dynamics. (**a**) A representative X-ray image acquired by high-speed X-ray imaging with depression zone, melt pool, spatter, and scan track highlighted. (**b**) Post-scan X-ray image with the location and geometry of melted scan track marked. (**c**) X-ray image with depression zone geometry indicated. (**d**) X-ray image with the spatter direction $\theta_{spatter}$ and velocity $V_{spatter}$ indicated.

Four features formed in the laser melting process were analyzed: depression zone, melt pool, spatter, and build track, as highlighted in a representative X-ray image in Figure 5a. Figure 5b highlights the shape and fluctuations in the scan track after laser scanning and solidification. Figure 5c demonstrates the depression zone location and geometry. Figure 5d outlines the spatter dynamics produced during SLM: spatter size, velocity, volume, and direction. Depression zone, melt pool, and spatter dynamics are 2D projections determined with in-situ high-speed imaging during selective laser melting of Ti6Al4V. Scan track height is determined from the X-ray images captured before and after laser scanning. Spatter diameter and volume are measured assuming a spherical geometry.

For analysis, the dimensions of the depression zone depth and width were extracted from the dynamic X-ray images and averaged for each powder sample. The scan track profile is determined after the melt pool solidifies. The spatter is determined by tracing the ejection of liquid spatter from the powder bed region. Once liquid spatter escaped from the powder region, the dynamics were analyzed. The angle of spatter is measured relative to laser scan direction. The projected velocity (on the imaging plane) of spatter is determined by dividing the moving distance of the spatter by the corresponding travel time of the spatter. The diameter of the liquid spatter is determined once the spatter leaves the powder bed region. The spatter’s diameter, d, is calculated by averaging the *x*-axis and *y*-axis diameter of the powder assuming a near-spherical shape. The volume of spatter is estimated by the summation of all spatters within the field of view, assuming all spatters are spherical. Equations for calculating the values are listed in the nomenclature section. Testing is conducted multiple times for each powder sample and the average value of all tests are reported here.

## 3. Results and Discussion

### 3.1. Avalanche Angle and Break Energy

**Figure 6:**
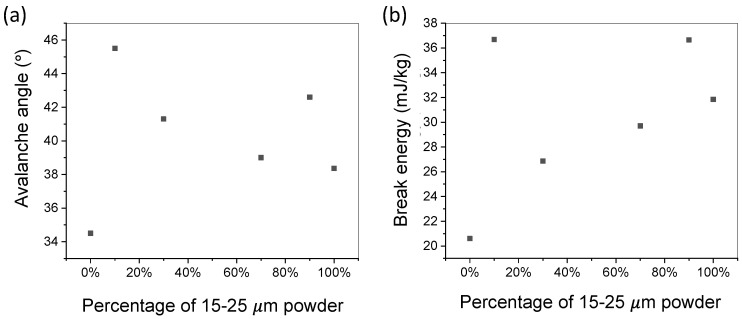
Avalanche angle and break energy. (**a**) Avalanche angle of powder samples with different percentages of 15–25 μm powder. (**b**) Break energy of powder samples with different percentages of 15–25 μm powder. Peaks are observed at the designed samples with efficient powder packing.

The avalanche angles of the powder samples measured by the revolution analyzer are shown in Figure 6a. Changing the powder size distribution from a homogeneous to non-homogeneous distribution (by mixing powders with different sizes) leads to an increase in the avalanche angle. This is caused by an increase of finer particles being introduced within larger powder systems, decreasing the overall flowability of the system by inducing inter-particle friction as described and demonstrated by Brika et al. [18]. Work from Liu et al. also highlighted reductions in flowability characteristics such as the Hausner ratio increase due to change from a near-homogenous to a non-homogeneous powder system [20]. From the testing results, the lowest avalanche angle was observed at the powder samples with 0% and 100% 15–25 μm powder. A unique trend was observed at the dense packing mixtures. At the dense packing samples with 10% and 90% 15–25 μm powder, a large increase in the avalanche angle was observed as compared to the surrounding powder samples. Within the powder system, the dense powder packing causes increased amounts of inter-particle friction [18]. The increase in frictional force resists the transition to kinetic avalanche during powder rotation, resulting in the decrease of the flowability of the powder samples with efficient packing.

The break energy of the six powder samples with different PSDs showed an upward trend as the amount of 15–25 μm powder increases. At the powder samples with high packing density (10% and 90% 15–25 μm powder), a large jump in the break energy was observed. The highest break energy observed is 37 mJ/kg at the sample with 10% 15–25 μm powder; the lowest break energy observed is 20.6 mJ/kg at the sample with 0% 15–25 μm powder. The most noticeable effect is the sudden jump of the break energy by 16 mJ/kg when the percentage of 15–25 μm powder increases from 0% to 10%. The cause of break energy increase is the increase of frictional contact between particles similar to the reason for the avalanche angle increase.

The detailed values of avalanche angle and break energy of the powder samples studied are listed in Table 3.

### 3.2. Effect of Powder Dense Packing on Flowability

Flowability testing results show that the flowability does not change linearly when adding 15–25 μm powder to 38–45 μm powder. However, sharp peaks are observed at the powder mixing ratios with efficient packing. The sharp decrease of flowability (indicated by the sharp increase of avalanche angle and break energy) at the powder mixing ratios with efficient packing may be attributed to the increase of cohesivity [27], internal friction [28], and local jamming [29].

The dense packing ratios occur at 10 and 90 percent 15–25 μm powder, which is only 10% away from the 38–45 μm powder and 15–25 μm powder, respectively. This indicates that the addition of small amount (10% for this instance) of non-uniform powder may lead to sudden drop of powder flowability, which can cause uncertainties in powder spreading process or jamming in powder delivery process. Determining the powder’s PSD to ensure that it is not near any powder dense packing ratio before loading it into an AM machine is important for avoiding powder delivery and spreading issues.

To mitigate or eliminate flowability uncertainty, reducing the powder size distribution to as close to homogenous as possible is preferred [20]. Since obtaining homogeneous powder is difficult or expensive for large scale production, reducing the distribution curve by eliminating the outlier powders will decrease the chance of getting powder dense packing ratios.

### 3.3. Selective Laser Melting Dynamics

**Figure 7:**
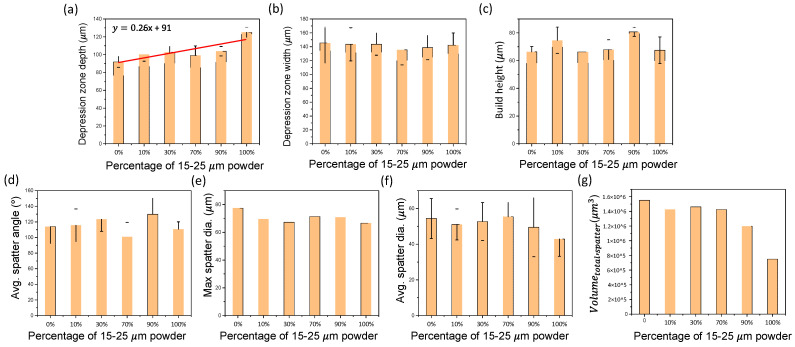
SLM dynamics. (**a**,**b**) Effects of PSD on depression zone depth and width. (**c**) Effects of PSD on build height. (**d**–**g**) Effects of PSD on spatter angle, maximum spatter diameter, average spatter diameter, and spatter volume.

Figure 7 shows the effects of particle size distribution on selective laser melting behavior (including depression zone, build height and spatter dynamics).

Figure 7a,b depicts the change in the depression zone depth and width due to varying the mixing ratio of small (15–25 μm) and large (38–45 μm) powders. An upward slope of 0.26 was observed as the percentage of small powder increases. The trend is due to the change in powder size, as the large powder requires greater energy input to melt and fuse powders to the substrate during scanning. The cause for the increased energy input is due to the reduction in laser absorption of the powder bed as the size of the powder increases, which is attributed to the decrease of multi-reflection induced absorption in the powder layer [30]. The depression zone width was not significantly affected by the powder size distribution.

The fluctuation of the depression zone depth and width of each powder sample during laser scanning was quantified by the standard deviation (indicated by error bars in Figure 7a,b), which does not show any trend.

Figure 7c depicts the effects of powder size distribution on the build height. A noticeable trend was found at the two designed mixing ratios with efficient powder packing. The samples with 10% small powder and 90% small powder exhibit high average build height with increases of 11% and 19% relative to the 100% 15–25 μm powder, respectively. Build height fluctuation was quantified by standard deviation (indicated by error bars in Figure 7c), which exhibits the smallest value at the sample with 90% small powder with an average deviation less than 5 μm.

Figure 7d–g demonstrates the effects of powder size distribution on spatter dynamics. No clear trend was observed for spatter angle and spatter size (maximum diameter and average diameter); a general decreasing trend is observed for spatter volume as the percentage of 15–25 μm powder increases during laser scanning. A 10% decrease of 15–25 μm powder from 100% to 90% caused the greatest increase of the spatter volume by almost 60%.

### 3.4. Effect of Powder Dense Packing on SLM Process

The influence of the powder dense packing on SLM process is mainly on the build height. The two designed powder mixtures with efficient packing, 10% 15–25 μm powder sample and 90% 15–25 μm sample, exhibit greater than 10% and 20% increase in the build height, respectively (processed with the same initial powder bed layer thickness). The powder mixtures with dense packing enable the deposition of a greater total mass of powder within a single layer on the build track. During laser scanning, more material is available to build the track to increase the build height. The increased height during scanning reduces the production time by reducing the number of layers required for part fabrication. Developing an effective method to obtain a dense powder bed using powder mixtures with efficient packing is a potential approach to improve productivity of metal AM.

## 4. Conclusions

This work investigated the effects of PSD on the flowability and SLM dynamics with the focus on the powders with efficient packing. The major results are summarized below.

- Powder flowability (characterized by avalanche angle and break energy) of six powder samples with different PSDs were reported. The changes of avalanche angle and break energy of up to 10.9° and 16 mJ/kg, respectively, were observed among the samples.
- The two designed powder mixtures with efficient powder packing lead to sudden increases in both the avalanche angle and break energy as compared to the surrounding PSDs by up to ~30% and ~70% increases, respectively, which is attributed to increased amounts of inter-particle friction.
- Effects of the PSDs on selective laser melting dynamics (depression zone, scan track, and spatter dynamics) were characterized and analyzed. A general decreasing trend is observed for spatter volume as the percentage of small powder increases during laser scanning.
- The designed powder mixture with efficient packing leads to an increase of build height of up to 20% as compared to 100% 15–25 µm powder processed with the same initial powder bed layer thickness.

## Figures and Tables

**Table 1:** Particle size distributions.

**Particle Size Distribution**	**Powder Samples (Indicated by Number Percentage of 15–25 μm Powder)**					
	**0%** **15–25 μm**	**10%** **15–25 μm**	**30%** **15–25 μm**	**70%** **15–25 μm**	**90%** **15–25 μm**	**100%** **15–25 μm**
$D_{10}$	22	15	12	12	11	7
$D_{30}$	32	23	16	16	14	10
$D_{50}$	38	30	20	20	17	13
$D_{70}$	41	39	25	24	20	17
$D_{90}$	45	44	31	28	25	22

**Table 2:** Density and Hausner ratio.

Powder Samples (Indicated by %15–25 μm powder)	Apparent Density, $\rho_{apparent}$ (g/cc)	Tapped Density, $\rho_{tapped}$ (g/cc)	Hausner Ratio, $\frac{\rho_{tapped}}{\rho_{apparent}}$
0	2.45	2.66	1.09
10	2.49	2.74	1.10
30	2.43	2.65	1.09
70	2.33	2.61	1.12
90	2.33	2.66	1.14
100	2.50	2.66	1.06

**Table 3:** Detailed values of avalanche angle and break energy.

Powder Samples (Indicated by %15–25 μm Powder)	Avalanche Angle (°Degrees)	Break Energy (mJ/kg)
0	35	21
10	46	37
30	41	27
70	39	30
90	43	37
100	39	31

## Data Availability

Data sharing is not applicable.

## Nomenclature

Symbol	Definition
$r_{i}$	Radius of central powder (μm)
$r_{j}$	Radius of surrounding powder (μm)
*R*	Ratio of central powder $r_{i}$ to surrounding powder $r_{j}$ ($R=\left(\frac{r_{i}}{r_{j}}\right)$)
$N^{T}$	The number of full (N) and partial $r_{j}$ powders that can theoretically be placed around central $r_{i}$ powder
N	Integer of $r_{j}$ powders that can theoretically be placed around central $r_{i}$ powder.
G	Gravitational constant $\left(\frac{m}{s^{2}}\right)$
$H_{pixel}$	Height of pixel (m)
$M_{total}$	Total mass (kg)
$V_{total}$	Total volume (cc)
$V_{pixel}$	Volume of pixel (cc)
$M_{pixel}$	Mass of pixel (kg)
d	Average diameter of ejected liquid spatter $d=\frac{d_{vertical}+d_{horizontal}}{2}$
$\theta_{spatter}$	Direction of ejecting spatter relative to the direction of the laser (left to right) and the substrate
$V_{spatter}$	Velocity of spatter projected on the imaging plane $V_{spatter}=\frac{\sqrt{{\left(Y_{2}-Y_{1}\right)}^{2}+{\left(X_{2}-X_{1}\right)}^{2}}}{t_{2}-t_{1}}$
$v_{spatter}$	Volume of all spatter produced during laser scanning $v_{spatter}=\overset{n}{\underset{i=1}{\sum }}\left(\left(\frac{\pi }{6}\right)d_{n}^{3}\right)$
$U_{pixel}$	The potential energy of a single powder pixel within the system
AM	Additive Manufacturing
SLM	Selective laser melting
SEM	Scanning electron microscope
$D_{10–90}$	Particle size distribution
